# Copper Promotes TFF1-Mediated *Helicobacter pylori* Colonization

**DOI:** 10.1371/journal.pone.0079455

**Published:** 2013-11-13

**Authors:** Sandro Montefusco, Roberta Esposito, Luca D’Andrea, Maria Chiara Monti, Ciara Dunne, Brendan Dolan, Alessandra Tosco, Liberato Marzullo, Marguerite Clyne

**Affiliations:** 1 Department of Pharmacy - Division of Biomedicine “Arturo Leone”, University of Salerno, Fisciano (SA), Italy; 2 Institute of Biostructures and Bioimaging, CNR, Naples, Italy; 3 Department of Pharmacy - Division of Chemistry and Chemical Technologies “Luigi Gomez Paloma”, University of Salerno, Fisciano (SA), Italy; 4 School of Medicine & Medical Science, University College Dublin, Dublin, Ireland; Vanderbilt University School of Medicine, United States of America

## Abstract

The trefoil peptides (TFF1, TFF2 and TFF3) are a family of small highly conserved proteins that play an essential role in epithelial regeneration within the gastrointestinal tract, where they are mainly expressed. TFF1 expression is strongly induced after mucosal injury and it has been proposed that *tff1* functions as a gastric tumor suppressor gene. Several studies confirm that *tff1* expression is frequently lost in gastric cancer because of deletions, mutations or methylation of the *tff1* promoter. Infection by *Helicobacter pylori* (*H. pylori*) results in chronic gastritis and it can lead to the development of gastric or duodenal ulcers. Moreover, it is known that there is a strong link to the development of gastric cancer. It has been shown that *H. pylori* interacts with the dimeric form of TFF1 and that the rough form of lipopolysaccharide mediates this interaction. We have previously reported that the carboxy-terminus of TFF1 is able to specifically bind copper ions (Cu) and that Cu binding favours the homodimerization of the peptide, thus enhancing its motogenic activity. Here, we report that the Cu-TFF1 cuprocomplex promotes adherence of *H. pylori* to epithelial cells. Adherence of *H. pylori* to gastric adenocarcinoma cells, AGS AC1 cells, induced to hyper-express TFF1 was enhanced compared to noninduced cells. Copper further promoted this interaction. A *H. pylori* mutant unable to bind TFF1 did not show enhanced infection of induced cells. Cu treatment induced a thickening of the mucus layer produced by the colorectal adenocarcinoma mucus secreting, goblet cells, HT29-E12 and promoted *H. pylori* colonisation. Finally, SPR analysis shows that the C-terminus of TFF1, involved in the binding of copper, is also able to selectively bind *H. pylori* RF-LPS.

## Introduction

Maintenance of the integrity of gastrointestinal tissue is physiologically essential in the presence of the persistent harassment of microbial flora and injurious agents. The repair of the gastric epithelium is modulated by several factors. One such factor is a family of small peptides called trefoil factors (TFFs). The trefoil factor family comprises the gastric peptide pS2/TFF1, the spasmolytic peptide (SP)/TFF2 and the intestinal trefoil factor (ITF)/TFF3; they are characterized by a three looped domain, the “trefoil domain”, stabilised by three disulphide bridges [1].

TFF1 expression is strongly induced after mucosal injury and it is involved in the restitution and regeneration processes of gastric mucosa [2]. Knock-out *tff1* mice develop gastric mucosa abnormalities and some mice develop multifocal intraepithelial or intramucosal carcinomas [3]. Therefore, it has been proposed that TFF1 functions as a gastric tumor suppressor gene. Moreover several studies confirm that *tff1* expression is frequently lost in cancer due to deletions, mutations or methylation of the *tff1* gene [4]–[6].

Gastric adenocarcinoma is the second leading cause of cancer-related death in the world [7]. Epidemiological studies in humans correlate *H. pylori* infection with peptic ulcers, gastric atrophy, distal gastric adenocarcinoma and other gastric diseases [8]. Consequently, it has been classified by the World Health Organization as a class 1 carcinogen.

*H. pylori* colonizes the gastric mucosa of humans and primates and it is one of the commonest infections of mankind. Infection is usually acquired during childhood, and when left untreated generally persists for the lifetime of the host [9]. The pathological infection is determined by multiple factors, including host genetic predisposition, *H. pylori* strain heterogeneity and environmental factors [10]–[11].

*H. pylori* shows a characteristic tropism for the mucus-producing gastric epithelium. The MUC5AC glycoprotein, carrier of the carbohydrate blood-group antigen Lewis B (Le^b^), is the primary receptor for *H. pylori* in the human stomach [12]. Moreover, it is well-known that MUC5AC interacts with TFF1 [13] and the two proteins are co-expressed in the stomach [14]. In addition, *H. pylori* interacts with the dimeric form of TFF1 [15] through its lipopolysaccharide (LPS) [16]. Finally, recent studies demonstrated that the interaction of TFF1 with *H. pylori* is important for colonization of gastric mucus and that HT29-E12 cells, a mucus secreting clone of HT29 colon carcinoma cells, are a useful system to study the interaction of bacteria with mucosal surfaces [17]. *H. pylori* co-localizes with TFF1 in both mucus layer of HT29-E12 cells and gastric biopsies [17].

We previously demonstrated that TFF1 is able to specifically bind copper ions at the carboxy-terminus [18] and that copper binding favours the homodimerization of the peptide, thus enhancing its motogenic activity [18]. The finding that Cu can influence expression [19], biological activity and structure of TFF1 prompted us to use Cu to investigate the effect of homodimerization of TFF1 on the interaction between *H. pylori* and the peptide. Here we demonstrate that the Cu-TFF1 complex promotes *H. pylori* colonization of gastric epithelial cells and of a mucus secreting cell line. Furthermore, we show that copper also promotes mucus layer formation in HT29-E12 cells. Finally, using Surface Plasmon Resonance we show that the C-terminus of TFF1, able to bind copper, is also involved in binding to *H. pylori* LPS.

## Materials and Methods

### Cell Culture

The HT29-E12 cell line, a mucus secreting subclone of the human colorectal adenocarcinoma cell line, HT29-MTX, was a generous gift from Professor Per Artursson, Uppsala University, Sweden. This clone was selected on the basis of tight junction formation and development of a mature adherent mucus layer [20]. HT29-E12 cells were maintained in Dulbecco’s Modified Eagle Medium (DMEM; Lonza) supplemented with 10% (v/v) FBS, 100 U/ml penicillin (Sigma), 100 µg/ml streptomycin (Sigma). For experiments, cells were grown for up to 21 days on trans-well filters 12 mm in diameter, with a 0.4 µm pore size (Millipore). Medium was replaced every two days.

The human clone hyperexpressing TFF1 under doxycycline induction (AGS-AC1) was selected from the human gastric cancer cell line AGS as previously described [18]. AGS-AC1 cells were cultured in DMEM medium supplemented with 10% (v/v) FBS, 100 U/ml penicillin, 100 µg/ml streptomycin (Sigma), and 600 µg/ml neomycin (Sigma). TFF1 expression was induced with 1 µg/ml of doxycycline.

### Bacterial Strains and Culture Conditions

*H. pylori* P12 strain is a typical clinical type-I isolate obtained from a patient with a duodenal ulcer and was previously described [21]–[22]. Frozen *H. pylori* stocks were recovered onto Columbia agar (Oxoid) containing 7% (v/v) defibrinated horse blood. Cloning and mutagenesis of isogenic mutant P12ΔHP1191 was as described previously by Dolan *et al*. [17]. This strain was constructed and donated by Prof Steffen Backert, Germany. *H. pylori* isogenic mutant strains were cultured in media supplemented with kanamycin sulphate (Fluka) to a final concentration of 10 µg/ml. All *H. pylori* strains were cultured at 37°C in gas jars (Oxoid; BBL) under microaerophilic conditions generated by CampyGen gas packs (Oxoid).

### Western Blot Analysis

AGS-AC1 cells were seeded at a density of 2×10^5^ cells/well in 6 well plates (φ 35 mm). TFF1 induction and *H. pylori* infection were carried out 24 and 48 hours respectively after cell seeding. Cell cultures were washed in PBS and fresh medium without doxycycline was added prior the infection. 50 µl of *H. pylori* suspension (OD_600_ = 1) were added per well, and incubated at 37°C for up to 5 hours, in microaerophilic conditions generated in gas jars by CampyGen gas packs. After incubation, cells were gently washed three times with sterile PBS and then lysed in 100 µl PBS, 1% Triton X-100, 2 mM Sodium Orthovanadate (Sigma). *H. pylori* lysates were prepared by harvesting the bacteria from blood agar plates and suspending then in PBS. Cell extracts were obtained by heating the bacterial suspension at 100°C, 10 min, in NuPAGE SDS sample buffer (Invitrogen).

Protein concentration was determined by Bradford assay and samples were analyzed by Western blot. Proteins were separated on an 8% SDS–PAGE and then electroblotted on a Hybond- ECL membrane (GE Healthcare) for 1 h, at 4°C and 100 V. Blots were probed with anti-CagA antibody (Austral Biologicals) and with anti-P-Tyr (4G10; Upstate Biotechnology).

### Measurement of IL-8 Production by AGS-ACI Cells upon Infection with *H. Pylori*

Interleukin-8 (IL-8) levels in uninfected and infected AGS-AC1 cells were determined using the Human IL-8 ELISA MAX™ Deluxe Kit (Biolegend). Supernatants from cells were collected following infection with *H. pylori*, clarified by centrifugation and stored at −80°C until use. ELISA’s were carried out in 96-well plates and absorbance was measured at 450 nm using SoftMax Pro software.

### Immunofluorescent Staining of AGS-AC1 Cells

Cells were seeded onto coverslips in multi-well dishes as described in the previous paragraph. After infection, coverslips were washed with PBS, fixed 10 minutes in 2% formalin and permeabilised with 0.2% (w/v) saponin (Sigma) in PBS for 10 minutes. Finally they were incubated for 1 hour in 1% (w/v) Bovine Serum Albumin (BSA; Sigma) and 10% (v/v) goat serum in PBS. Coverslips were then incubated overnight with antibodies against TFF1 [23], *H. pylori or* Lewis^b^ blood group antigen, (SPM194, Santa Cruz) at 4°C in a humidified atmosphere. After washing with PBS, they were incubated with an Alexafluor 488-conjugated anti-mouse antibody (Invitrogen), or Alexafluor 594-conjugated anti-rabbit antibody. To counterstain the nuclei, DAPI (Invitrogen) was added along with the secondary antibody. Coverslips were mounted onto glass slides and examined using a fluorescent microscope.

### Infection Assays

AGS-AC1 cells were seeded in 12-well plates at a density of 5×10^4^ cells in Ham’s F12 supplemented with 600 µg/ml neomycin. Twenty-four hours later, media was changed to Ham’s F12 and the cells were induced with 1 µg/ml doxycycline, 100 µM copper or 500 µM copper chelator bathocuproine disulfonate (BCS), or both for 48 hours after which time the cells were washed three times with PBS to remove any traces of doxycycline and copper, then to each well, 200 µl of Ham’s F12 and 100 µl of *H. pylori* (O.D_600_ of 0.4) were added. Infected cells were incubated under microaerophilic conditions at 37°C for 2 hours. Following infection, cells were washed with PBS to remove any non-adherent bacteria and then harvested using trypsin EDTA. Serial dilutions of the cells were plated out in duplicate on Columbia blood agar plates in order to enumerate the number of bacteria associated with the cells. Plates were incubated at 37°C under microaerophilic conditions. Following 4–5 days incubation, the bacterial colonies were counted and the number of colony forming units (C.F.U)/ml was calculated.

HT29-E12 cells were grown on transwell filters as described above. 24 h before infection, antibiotic containing medium was removed, monolayers were gently rinsed with antibiotic free media and cells were incubated in antibiotic free media for 24 h in the presence of copper (CuCl_2_ 100 µM) or copper chelator, (BCS 500 µM). Bacteria were grown as described above and harvested from Columbia blood agar into BHI broth at pH 5.0. Media in the lower chamber of the transwell was replaced with 1.5 ml of sterile antibiotic free DMEM supplemented with 10% FBS. Since *H. pylori* is not able to survive in DMEM, medium in the upper chamber of the transwell was replaced with 100 µl of sterile BHI broth, pH 5.0, in the presence of copper salt or copper chelator. It has been previously shown that these conditions are able to support *H. pylori* growth and binding to polarized epithelial cells without any adverse effect on cell growth [24]. 50 µl of *H. pylori* suspension, OD_600_ = 0.4 were then added to the upper chamber of the transwell, and infected cell cultures were incubated at 37°C under microaerophilic conditions up to 24 hours. Cultures were gently washed with sterile PBS on both sides of the transwell and cells were harvested after trypsin/EDTA treatment as previously described by Cottet *et al*. [24]. Serial dilutions of the cells were plated out in triplicate onto Columbia blood agar plates and incubated at 37°C in gas jars under microaerophilic conditions. Colonies were counted after 4–5 days of incubation.

### Immunofluorescent Staining of HT29-E12 Cells

HT29-E12 cells treated with CuCl_2_ 100 µM, BCS 500 µM or untreated cells growing on transwell filters were gently washed with sterile PBS. Filters were then removed from their plastic supports and sandwiched among two thin slices of chicken liver prior to mounting in Optimal Cutting Temperature (OCT) medium (BDH) as described by Keely and coworkers [25]. Sections (16 µm thick) of the OCT mounted cultures were cut using a cryostat (Leica), collected onto polylysine coated microscope slides and allowed to air dry for 10–15 minutes. Sections were then immediately stained or stored at −20°C. Briefly, they were fixed in 2% formalin for 10 minutes and permeabilised with 0.2% saponin in PBS for 10 minutes. Blocking was performed for 1 hour in PBS containing 1% Bovine Serum Albumin (BSA; Sigma) and 10% goat serum. Sections were then incubated overnight with antibodies against TFF1 [23] or MUC5AC C-terminus (45M1, Sigma), at 4°C in a humidified atmosphere, washed with PBS and subsequently incubated with appropriate secondary antibodies. Nuclei were counterstained by adding DAPI (Invitrogen) into the secondary antibody solutions. Coverslips were mounted on the slides using Fluorescent Mounting Medium (Dako) and the sections were examined using a fluorescent microscope.

### Alcian Blue/Neutral Red Staining of HT29-E12 Cells

In order to visualise the adherent mucus layer present on cells, HT29-E12 frozen sections obtained as described above, were stained with Alcian Blue/Neutral Red. Sections were incubated 5 min at room temperature with Alcian Blue (0.5% Alcian Blue 8GX, 3% acetic acid) washed 5 min with water and incubated, 30 sec with Neutral Red (3.3 g/L in Dulbecco’s Phosphate Buffered Saline, Sigma) then washed again and mounted on coverslips.

### Statistical Analysis

Experiments were repeated at least three times, as technical triplicates. Results are reported as mean values ± standard deviations (error bars) of the replicated experiments. The Student *t* test was used to estimate statistical significance.

### Peptide Synthesis and Purification

In order to identify the region of TFF1 involved in the binding with *H. pylori* LPS, SPR assays were set up to test the binding ability of the C-terminal region of the protein, also involved in the binding of copper ions. Peptides were synthesized and purified as previously described [18]. Peptides were biotinylated (Bt) on resin, after removal of N-terminal 9-Fluorenylmethoxycarbonyl (Fmoc) protecting group, using 5 eq of N-(+)-Biotinyl-6-aminocaproic acid (Sigma Aldric), 5eq of N-[(Dimethylamino)-1H-1,2,3-triazolo-[4,5-b]pyridin-1-ylmethylene]-N-methylmethanaminium hexafluorophosphate N-oxide (HATU, Inbios) and 10 eq of N,N-Diisopropylethylamine (DIPEA, Romil) in N,N-Dimethylformamide (Romil) over night at room temperature. Peptides were cleaved from the resin with trifluoroacetic acid (TFA, Romil)/triisopropyl silane (TIS, sigma)/water (95∶2.5∶2.5 v/v/v) mixture for 3 hrs at room temperature. Peptide analyses and purification were performed by RP-HPLC on a C18 Jupiter column (Phenomenex), and their identity was assessed by ESI mass spectrometry on Thermo Finnigan MSQ LC–MS.5_70_20.

pTFF: Bt-FYPNTIDVPPEEECEF-COOH; MW_calc_ 2,268.5 Da; found [M+2H^+^]^2+^1134.4.

pSCR: Bt- EPNCTPIFFEPEDYVE-COOH; MW_calc_ 2,268.5 Da; found [M+2H^+^]^2+^1134.2.

Biotinylated peptides were incubated over night in a 10 molar excess solution of CuCl_2_ at 4°C to obtain peptide dimers. Monomer and dimer solutions were further purified by RP-HPLC on a C18 column eluted with acetonitrile/water/trifluoroacetic acid (95%/5%/0.07%), 5–85% linear gradient. Peptide masses were assessed by MALDI-MS performed on a MALDI-micro MX (Waters).

### LPS Binding Assay by Surface Plasmon Resonance

We assessed if the C-terminal region of TFF1 which is involved in the binding of copper ions also plays a role in binding to *H. pylori* LPS. LPS binding assays were performed by Surface Plasmon Resonance on a Biacore 3000 instrument (GE Healthcare). About 500 RU of biotinylated peptides (monomeric pTFF, dimeric pTFF and pSCR) were immobilized on a SA chip in HBS-P buffer (10 mM Hepes pH 7.4, 150 mM NaCl, 0.005% v/v Surfactant P20 - GE Healthcare) at a flow rate of 10 µl/min. Assays were performed at 25°C and the signal obtained from an empty flow cell was referred to as a baseline for all the experiments. *H. pylori* LPS prepared according to Dolan *et al.* [17] was injected at 5 increasing concentrations (30 µl/min for 2 min), while *E.coli* LPS was used as a control reference.

## Results

### Adherence of *H. Pylori* to AGS-AC1 Cells

**Figure 1:**
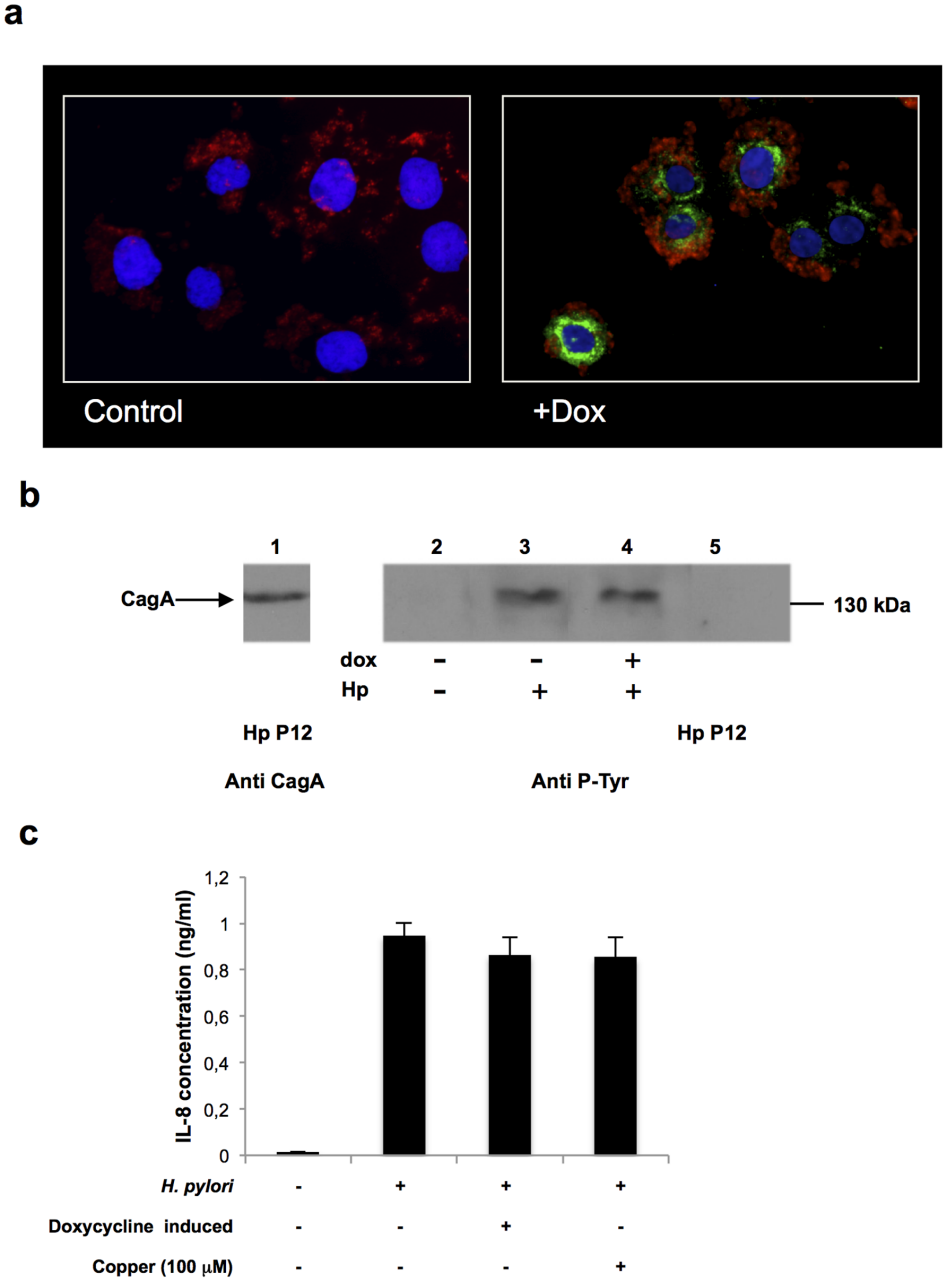
The interaction of *H. pylori* with AGS-AC1 cells. **a**: Immunofluorescent staining of *H. pylori* colonized AGS-AC1 cells. Induced and not induced cells were infected with *H. pylori*. Blue: DAPI staining for nuclei; Red: *H. pylori*; Green: TFF1. **b**: CagA phosphorylation following *H. pylori* colonization of AGS-AC1 cells. Western immunoblot was probed with anti-CagA and anti-phosphotyrosine antibodies. Lane 1: *H. pylori* P12 bacterial cell lysate probed with anti-CagA. Lanes 2–5 were probed with anti-phosphotyrosine antibodies. Lane 2: *H. pylori* P12 bacterial cell lysate; Lane 3: AGS-AC1 cells colonised with *H. pylori* P12; Lane 4: whole cell lysate of induced AGS-AC1 cells colonized with *H. pylori*; Lane 5: AGS-AC1 whole cell lysate. **c:** IL-8 production by uninduced and induced AGS-AC1 cells upon colonization by *H. pylori*.

To study the influence of the cuprocomplex TFF1-Cu on *H. pylori* colonisation, we used an inducible clone of the gastric carcinoma cell line AGS able to hyper-express TFF1, AGS-AC1 cells [18]. We evaluated the toxicity of Cu^2+^, BCS and Doxycycline on *H. pylori.* Doxycycline was toxic to the bacteria, hence we removed it from the cell culture medium prior to infection of the cells. Copper and BCS at the concentrations used had no effect on the viability of the organism (results not shown). A previous study has shown that *H. pylori* is resistant to copper concentrations as high as 500 µm [26]. Immunofluoresent microscopy demonstrated that the bacteria interacted with induced and uninduced AGS-ACI cells (Fig. 1a). Phosphorylated CagA was detected in TFF1 expressing and non TFF1 expressing cells indicating that molecular cross talk was occurring between the bacteria and the cells (Fig. 1b). TFF1 expression in the cells did not alter IL-8 production upon H. pylori infection (Fig. 1c).

### TFF1-Cu Complex Promotes *H. Pylori* Colonization of AGS-AC1 Cells

We used quantitative bacterial colonization assays to investigate the effect of TFF1 expression on *H. pylori* infection of AGS-AC1 cells. We also looked at the effect obtained by pretreating the cells with copper or copper chelator. Induced and uninduced AGS-AC1 cells were treated with copper (CuCl_2_ 100 µm) or copper chelator (BCS 500 µM) for 48 hr prior to infection.

**Figure 2:**
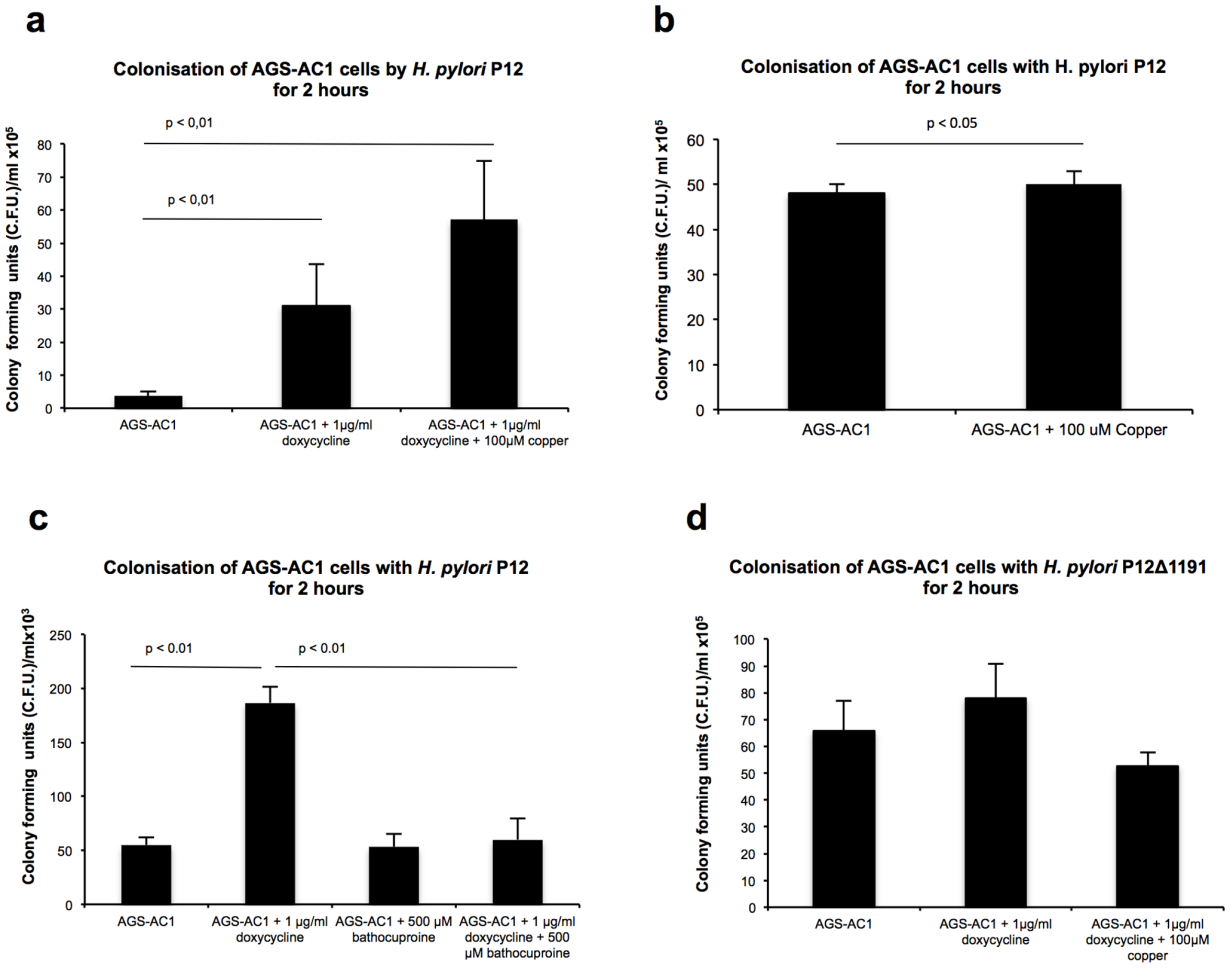
The effect of TFF1 and copper on *H. pylori* colonization of AGS-AC1 cells. AGS-AC1 cells, induced and uninduced, were infected with wild type strain (P12), or mutant strain P12ΔHP1191. The infections were carried out in the presence of Cu 100 µM or BCS 500 µM. Adherence was evaluated in comparison to control untreated AGS-AC1 cells. **a:** Effects of TFF1 and copper on *H. pylori* P12 cell adhesion. **b:** Effects of copper on *H. pylori* P12 cell adhesion. **c:** Effects of TFF1 and/or BCS on *H. pylori* P12 cell adhesion. **d:** Effects of TFF1 and/or copper on *H. pylori* P12ΔHP1191 cell adhesion.

The numbers of bacteria associated with induced cells overexpressing TFF1 was significantly higher than the numbers associated with uninduced cells. Moreover, pretreatment with CuCl_2_ resulted in a further increase in the number of bacteria colonising the TFF1 overexpressing cells (Fig. 2a), Copper had no effect on bacterial colonization of uninduced cells which did not express TFF1 (Fig. 2b) On the other hand, pretreatment with BCS abolishes the increase of cell associated bacteria observed in induced cells overexpressing TFF1 (Fig. 2c).

In order to further investigate if the presence of TFF1, and/or its cuprocomplexes, could affect bacterial adhesion to epithelial cells, we used an isogenic *H. pylori* mutant strain P12ΔHP1191. In this strain the lipopolysaccharide structure is modified [17]. Briefly, HP1191, encodes an LD-heptosyltransferase, which has been shown to be involved in the biosynthesis of the inner core region of the oligosaccharide portion of *H. pylori* LPS [27]. Insertional inactivation of HP1191 results in the production of LPS with a severely truncated core oligosaccharide. Dolan *et al.* [17] demonstrated that dimeric TFF1 is able to bind the LPS of P12 wild type but it fails to bind to LPS synthesized by the P12ΔHP1191. The additive effect of TFF1 and copper on the ability of *H. pylori* to colonize AGS-AC1 cells was absent when the P12ΔHP1191 mutant, which cannot bind TFF1, was used (Fig. 2d).

These results suggest that the effect of copper on colonization by *H. pylori* is related to the presence of TFF1 and probably to its influence on the structure of the peptide and on the equilibrium of its oligomeric forms [18].

### TFF1-Cu Complex Increases *H. Pylori* Colonisation of HT29-E12 Cells

**Figure 3:**
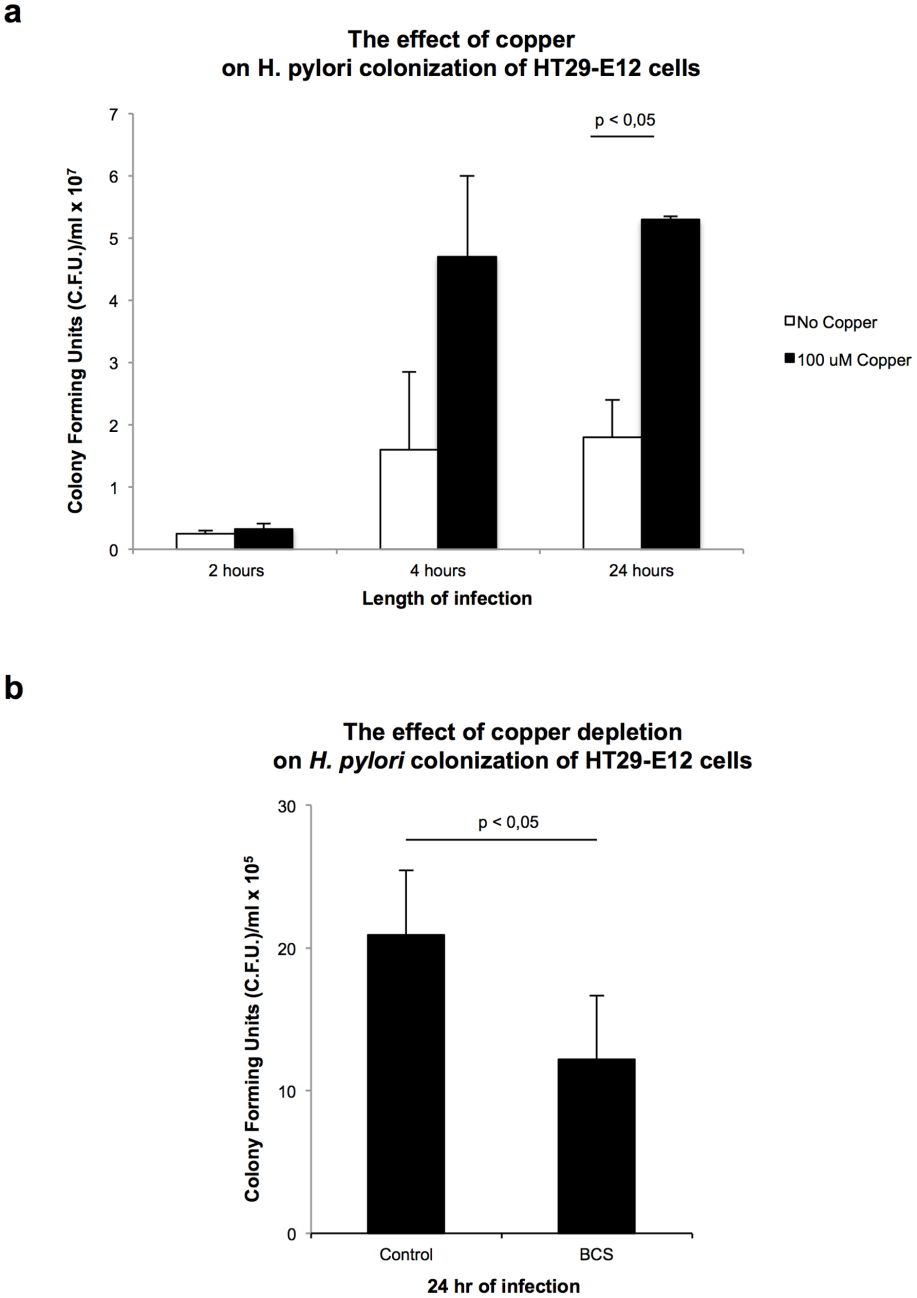
The effect of copper on *H. pylori* colonization of HT29-E12 cells. Colonization of HT29-E12 cells by *H. pylori* P12 in the presence of excess copper (Cu 100 µM; 2, 4, and 24 hr) (**a**) and in copper depleted conditions (BCS 500 µM; 24 hr) (**b**).

The HT29-E12 cell line is a suitable *in vitro* model to study the physiological role of the adherent mucus layer that covers many epithelial surfaces. Physico-chemical properties of the mucus in the gastrointestinal tract depend on its characteristic association of specific mucins and trefoil factors. HT29-E12 cells constitutively express TFF1 and the mucin MUC5AC which are excreted from the cells and contribute to form a mature adherent mucus layer after 21 days of culture [17]. *H. pylori* infection was assessed in copper overload or copper deficiency conditions. Exposure to CuCl_2_ (100 µM) resulted in an increase in *H. pylori* colonization after 4 hours and this effect was most pronounced after 24 hours when compared to control cells which had not been exposed to copper (Fig. 3a), while copper chelation with BCS 500 µM decreased the number of bacteria infecting the cells by about 40% (Fig. 3b). These results with HT29-E12 cells corroborates the observations made above with AGS-AC1 cells, that copper can promote colonisation of *H. pylori* in the presence of TFF1.

### Copper Promotes Mucus Layer Formation of HT29-E12 Cell Line

Our previous findings showed that copper promotes the dimerization of TFF1 [18] and that *H. pylori* binds TFF1 dimers [15]. The dimeric form of TFF1 is the predominant form associated with MUC5AC in the mucus [13]. On the basis of this evidence we hypothesized that copper could influence the formation of mucus and we examined the effect of copper on mucus layer formation in HT29-E12 cells.

**Figure 4:**
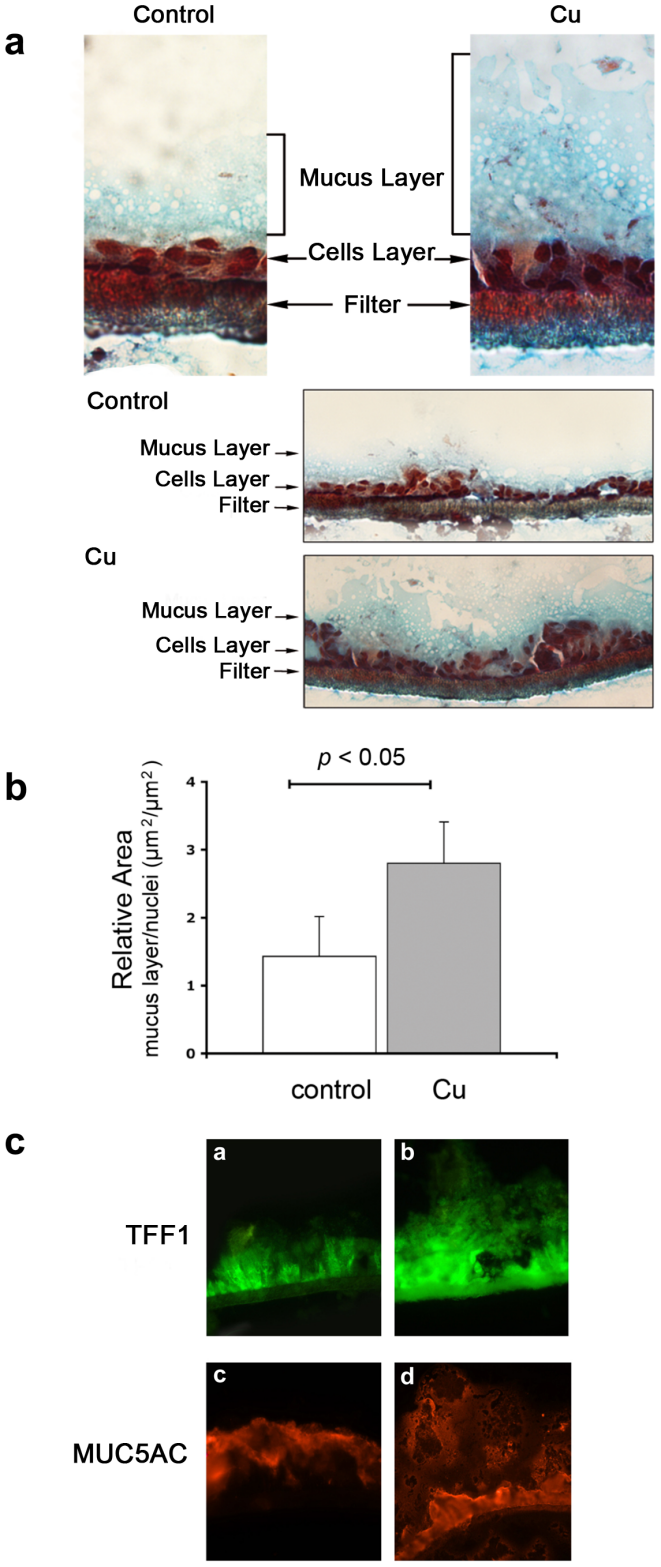
The effect of copper on mucus thickness in HT29-E12 cells. **a**: Alcian Blue/Neutral Red staining of HT29-E12 cells grown on trans-well filters. Cells were treated or not treated (control) with CuCl_2_ 10 µM, 48 hours. Staining shows the mucous secretions (blue) and nuclei of the cell monolayer (red). **b**: The graph shows the ratio between the areas of mucous secretions and nuclei, in control and treated samples. Values represent means +/− SD. **c**: Immunofluorescence analysis of TFF1 and MUC5AC expression in HT29-E12 cells cultured on trans-well filters. Staining of filter cross sections are shown. a, b: anti-TFF1 antibody. c,d: anti-MUC5AC antibody.

Examination of cells grown in the presence and absence of copper and subsequently stained for TFF1 and MUC5AC demonstrated that treatment with copper results in a thicker mucus layer where MUC5AC and TFF1 are clearly present (Fig. 4c). Furthermore, selective staining Alcian Blue/Neutral Red of muco−/glyco-proteins (pale blue) and cell nuclei (red) highlights the boundary of the mucus, thus making the thicker layer present on the top of copper treated cell (Fig. 4a–b). A visible increase in the thickness of the mucus layer could explain the increase in the number of bacteria associated with the cells due to a concomitant increase in the binding ability of the mucus layer.

### TFF1 Carboxy-terminal Tail is Involved in the Interaction with *H. Pylori* LPS

**Figure 5:**
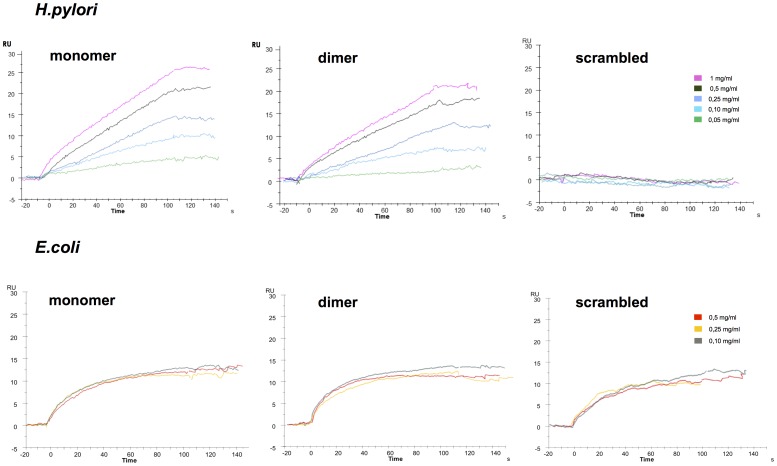
SPR analysis of *H. pylori* RF-LPS binding to TFF1 C-terminus. The biotinylated synthetic peptides are composed of the last 16 aa of native TFF1. Panel A, B, C show the analysis of peptide monomers, dimers and scrambled monomers respectively challenged with *H. pylori* RF-LPS. Panels D, E, F show the analysis of peptide monomers, dimers and scrambled monomers respectively challenged with *E. coli* RF-LPS.

The above results suggest that the increase in bacterial colonization seen in the presence of copper depends on the expression of TFF1 and the formation of its homodimers which have been shown previously to be able to bind to the bacterial cell wall lipopolysaccharide [16]. The flexible tail of TFF1, formed by the last 16 aa at the C-terminus of the protein, is able to gain structure upon the addition of copper [28]. The positive and synergic effect of TFF1 and copper on *H. pylori* colonization led us to hypothesize that a possible improvement of LPS binding affinity, or a faster shift of the equilibrium towards the formation of TFF1 homodimers, could explain the higher rate and yield of bacterial colonization observed in the presence of both inducers. To characterize the binding of *H. pylori* LPS we carried out SPR binding assays using synthetic (N)-biotinylated TFF1 monomer and dimer peptides which represent the native sequence of the TFF1 C-terminus (16 aa) and a corresponding scrambled sequence. Both peptides were incubated in 10 molar excess of divalent copper cations to induce their dimerization. MALDI-MS analysis showed that copper treatment and chromatographic separation allowed pure monomers and dimers with the native sequences to be obtained. Only monomers of the scrambled sequence were obtained (data not shown). Peptides injected over separate flow cells were immobilized onto an SA chip (500 RU bound) and challenged with *H. pylori* LPS. SPR experiments showed an increase of LPS binding to the chip surfaces derivatized both with monomer and dimers proportional to the amount of injected LPS, (Fig. 5 A–B) while no SPR signal increase was observed in the scrambled peptide flow cell (Fig. 5 C). Control experiments carried out by challenging *E. coli* LPS on TFF1 and scrambled peptides confirmed the binding selectivity of the TFF1 sequences for *H. pylori* LPS. The experimental evidence shows that the TFF1 C-terminus could be the epitope present on TFF1 that mediates binding to *H. pylori* LPS, but does not explain the observed selective affinity of native protein dimers for LPS. It is thus possible that the bulk of the native protein could be involved and could modulate the access of LPS to the C-terminus.

## Discussion

The interaction of *H. pylori* LPS with TFF1 is likely to play a role in mediating bacterial colonization of the gastric mucosa [16]–[17]. The mucus layer acts as a reservoir close to the epithelial surface for the majority of the colonizing bacteria, where its physico-chemical properties allow for an environment that is more permissive for *H. pylori* compared to the gastric lumen and enables the bacteria to interact with the epithelium and subsequently cause disease.

The dimeric form of TFF1 is the biologically most active form of the protein. It acts as a motogen and plays a role in epithelial restitution when damage occurs. It is also the form able to bind the core oligosaccharide of *H. pylori* LPS. Our previous results showed that copper influences the balance of TFF1 oligomeric forms, promoting TFF1 dimerization *in vitro* [18]. Since it could be inferred that the shift towards the dimeric form should have functional consequences *in vivo*, experimental evidence prompted us to study the possible role of copper in promotion of bacterial colonization. We observed that, copper is able to improve the level of colonisation that occurs with AGS-AC1 cells expressing TFF1. Control experiments in the presence of basal levels of TFF1 and/or the addition of copper chelator showed that copper alone was not able to modify the yield of infection, but only showed a positive synergistic effect in the presence of TFF1. Thus copper could influence the local environment, and consequently *H. pylori* colonisation, presumably through the induction of TFF1 dimerization in the mucus. The effect of copper on the level of colonisation was also confirmed by colonisation assays using *H. pylori* mutants with a truncated core oligosaccharide. The results show that no effect could be observed when the *H. pylori* P12ΔHP1191 mutant, expressing severely truncated LPS unable to bind TFF1, was used to infect the cells. Results suggest that copper can modulate the TFF1/LPS interaction and influence bacterial colonisation.

Furthermore, experiments carried out with HT-29-E12 cells, which secrete an adherent mucus layer, showed that copper treatment influences the thickness of the mucus layer. This evidence could provide an explanation for the increased bacterial colonisation seen with these cells in the presence of copper as an increase in the volume of the mucus layer means there is a concomitant increase in bacterial binding sites close to the cell surface. On the other hand it is worth noting that the increase of mucus thickness *in vitro* reproduces a static configuration of the mucus layer, which *in vivo* is naturally mobilised by the physiological motility of gastric walls or by the intestinal peristalsis, mainly in its uppermost luminal layer subject to mechanical shedding.

Further analyses of the rheological properties of the mucus will clarify if the level of copper can modulate its thickness and viscosity and foster colonisation or improve the clearance of bacteria on the gastrointestinal epithelium. Mucus covering epithelial cells, behaves as a pseudo-plastic fluid whose rheological properties depend on the specific association of mucins and trefoil peptides Previous papers reported the direct association of MUC5AC and TFF1 [13] and their coordinated gene expression and localization [14]. In addition other authors concluded that trefoil peptides exert their protective and healing functions through their ability to catalyse the formation of stable mucin complexes [29]. Shear-thinning properties of mucous gels are modulated by MUCs/TFFs specific combinations and are probably correlated to their localization and functions. Indeed, a low viscosity mucus (containing TFF1 or TFF3) is more useful on epithelia where mucus flow leads to clearance of germs and foreign particles that get trapped in the mucus on their surfaces (intestine, respiratory tract, eyes, genitalia, etc.), while high viscosity mucus (containing TFF2) stabilizes a more efficient and protective shield for epithelial surfaces subject to chemical and physical injuries (stomach). An increase of thickness and low viscosity of TFF1 containing mucus could favour *in vivo* the clearing of mucosa by faster removal of bacteria trapped in the mucus. An epidemiological study on food intake and *H. pylori* infection [30] suggested a possible effect of dietary intake and serum concentration of zinc and iron on the bacterial infection, but no conclusive correlation could be found with dietary copper except for a slightly higher serum concentration of the metal in positive subjects. Further analyses of rheological properties of the mucus and further investigation following dietary copper supplementation *in vivo* could provide useful experimental evidence on the possible effects of the metal in combination with standard therapies.

Preliminary SPR results obtained with synthetic peptides representing the C-terminal tail of TFF1 show that this crucial region of the protein is involved in the selective binding of *H. pylori* LPS, but it cannot explain the selectivity of the bacterium for native TFF1 dimers. Structural analysis to characterize the LPS/TFF1 complexes could clarify the role of copper and its ability to influence the structure and provide further insights on the molecular mechanism used by H. pylori to interact with LPS thus enabling the development of more selective and targeted tools to prevent colonization.

Finally, the increase in bacterial adhesion induced by TFF1 and copper in AGS-AC1 cells, unable to produce mucus, poses a further unresolved question about possible and more complex roles of the peptide in host infection. Although TFF1 has been shown on the plasma membrane of MCF-7 cells [31], several attempts aimed at identifying possible TFF1 receptors and/or protein partners on the plasma membrane of epithelial cells have been unsuccessful. A better understanding of molecular mechanisms able to influence the onset of colonisation and survival of *H. pylori* through binding to ligands present in the mucus could provide novel targets to further modify and improve the strategies to attenuate bacterial virulence and support the use of antibiotics.
